# Bioengineering perspectives on adhesive delivery: performance analysis of dental microapplicators

**DOI:** 10.3389/fdmed.2025.1698820

**Published:** 2025-11-07

**Authors:** Rim Bourgi, Celso Afonso Klein Junior, Laura Rebelo Allram, Luciana Gonçalves Heck, Louis Hardan, Carlos Enrique Cuevas-Suárez, Chana Mesquita, Fabio Herrmann Coelho-de-Souza, Naji Kharouf, Youssef Haikel

**Affiliations:** 1 Department of Restorative and Esthetic Dentistry, Faculty of Dental Medicine, Saint-Joseph University of Beirut, Beirut, Lebanon; 2 Department of Biomaterials and Bioengineering, INSERM UMR_S 1121, University of Strasbourg, Strasbourg, France; 3 Department of Restorative Sciences, Faculty of Dentistry, Beirut Arab University, Beirut, Lebanon; 4 Postgraduate Program in Dentistry, Universidade Luterana do Brasil (ULBRA), Canoas, Rio Grande do Sul, Brazil; 5 School of Dentistry, Universidade Luterana do Brasil (ULBRA), Canoas, Rio Grande do Sul, Brazil; 6 Dental Materials Laboratory, Academic Area of Dentistry, Autonomous University of Hidalgo State, San Agustín Tlaxiaca, Mexico; 7 Department of Dentistry, Universidade Federal do Rio Grande do Sul, Porto Alegre, Brazil; 8 Department of Endodontics and Conservative Dentistry, Faculty of Dental Medicine, University of Strasbourg, Strasbourg, France; 9 Pôle de Médecine et Chirurgie Bucco-Dentaire, Hôpital Civil, Hôpitaux Universitaire de Strasbourg, Strasbourg, France

**Keywords:** adhesive system, microapplicators, restorative dentistry, bonding (D), adhesives

## Abstract

**Background:**

The precise and consistent application of adhesive systems is essential for achieving reliable bonding in restorative dentistry. Microapplicators are commonly used for adhesive delivery; however, variations in their structural quality and performance may affect clinical outcomes.

**Objective:**

This study evaluated four commercially available microapplicator brands—Angelus, FGM, KG Sorensen, and SDI—regarding the quality of their active tips (bristle configuration) and adhesive delivery capacity.

**Methods:**

A total of 160 microapplicators (40 per brand) were analyzed. Optical microscopy (15 per brand) assessed bristle integrity before and after use. Scanning electron microscopy (SEM; 10 per brand) evaluated surface morphology (five unused and five used applicators per brand). Adhesive release capacity (15 per brand) was determined by weighing each microapplicator before adhesive loading, after loading, and following application to a standardized cavity. Data was analyzed using One-way analysis of variance (ANOVA).

**Results:**

New microapplicators from Angelus, KG Sorensen, and SDI displayed uniform bristle arrangements without visible gaps. After use, SDI and Angelus maintained superior bristle cohesion, whereas FGM showed the greatest deformation and sparse bristle distribution. Despite morphological differences, all brands delivered adhesive with comparable efficiency, exceeding 96% release.

**Conclusions:**

SDI microapplicators demonstrated the highest structural stability and resistance to deformation, followed by Angelus, KG Sorensen, and FGM. Although adhesive release capacity was consistent across all brands, differences in bristle quality may influence handling characteristics and clinical precision.

## 1 Introduction

In recent decades, the techniques and materials used in adhesive procedures have undergone major improvements (1–4). With the adhesive approach, it is possible to perform minimally invasive procedures or more conservative preparation designs based on the reliability and durability of the adhesive systems with the enamel and dentin (5). This procedure is highly operator- and instrument-dependent due to the numerous steps and protocols involved (6).

The application strategy of adhesive systems plays a critical role in determining the quality of the bonding layer on dentin. Smearing and actively agitating the adhesive with a microapplicator on the dentin surface enhance the interaction between the adhesive and the substrate, thereby improving the quality of the bond and extending the longevity of the restoration (7–10).

The use of additional application strategies can enhance the longevity and long-term performance of adhesive systems. However, the effectiveness of these strategies is highly dependent on the instrument used for application. Prolonged application time combined with active scrubbing improves adhesive infiltration and bonding to dentin. These factors—application time and intensity of scrubbing—are influenced by the quality and design of the microapplicator used (11, 12).

In the past, adhesive systems were applied to tooth surfaces using basic tools such as cotton pellets, tissue paper, sponge applicators, or disposable brushes with elongated bristles (13). However, in the early 2000 s, a more advanced and efficient tool—the microbrush—was introduced to the dental field (14). A microbrush is a disposable, hand-held applicator composed of a plastic handle with a fine nylon brush or microfibers (typically made of nylon, cotton, or synthetic polymer fibers) attached to one end (15). This design allows for greater precision and ease in applying various dental materials, such as adhesives, varnishes, etchants, and resins (16).

Microbrushes, often referred to as microapplicators, have become essential in adhesive dentistry due to their ability to load and deliver adhesive agents effectively. More importantly, they enable active application techniques, such as rubbing or smearing, which have been shown to enhance monomer penetration and bond strength (15). Active application, particularly of the primer component, significantly improves the interaction with dentin, leading to more durable and micromechanically stable bonds compared to passive techniques (10).

Previous research has demonstrated that using microbrushes as carriers for priming adhesive solutions results in more uniform and stable bonding mechanisms, particularly for procedures such as endodontic post cementation (17, 18). For current adhesive systems—which often require extended application times and vigorous rubbing or vibratory motion on dentin—microapplicators must also offer sufficient resistance at the tip. This ensures the applicator maintains its shape without excessive deformation or fiber loss during use (10, 19).

Ultimately, the quality of the microapplicator plays a crucial role in the effectiveness of adhesive application (20). Efficient bonding to the dentin structure depends not only on the chemistry of the adhesive system but also on the ability of the microapplicator to deliver and agitate the adhesive over the dentin surface without compromising performance (10, 13, 19).

However, limited research has been conducted on the presence of residual brush fibers left on the adhesive surface (13), as well as the potential deformation of the brush head during application. These factors are clinically relevant, as both the presence of impurities and the structural integrity of the applicator may compromise the quantity and quality of adhesive application and sealing—particularly at critical areas such as the cervical margin.

Therefore, the aim of this study was to evaluate the adhesive-carrying capacity of four different commercial brands of microapplicators and to assess their quality by analyzing the configuration and integrity of the bristles in the active tip, both before and after use on dentin.

## 2 Materials and methods

For this study, fifteen bovine teeth and medium-sized microapplicators of dental adhesives from four different brands (Angelus; FGM, KGSorensen and SDI) were used. Bovine teeth were selected as substitutes for human teeth due to their easier availability, ethical acceptability, and similar microhardness and mineral composition to human enamel and dentin. The four brands of microapplicators were selected based on their widespread use in clinical practice and their distinct fiber density, tip structure, and handle rigidity—factors that can influence adhesive delivery efficiency and reproducibility. This study did not involve the use of animals or live animal organs. Extracted cattle teeth, obtained as by-products from a meatpacking plant, were utilized. These teeth had already been employed in a previous published study (20). As such, approval from an animal ethics committee was not required.

The number of specimens per group (*n* = 15) was selected based on feasibility and consistency with a prior study evaluating adhesive performance (21). This sample size was considered adequate to achieve acceptable statistical power for detecting intergroup differences at a 5% significance level.

All applicators were purchased online from a dental store in the state of Santa Catarina (Dental Speed). To evaluate the quality of the microapplicators, optical microscopy and electron microscopy were used, comparing the image before using the microapplicator with the image after using the applicator. To evaluate the adhesive carrying capacity, a precision analytical balance was used, as described below (Figures 1–16).

### 2.1 Tooth collection, cleaning, storage and cavity preparation

Fifteen bovine teeth were used for the study. Before being prepared, the teeth were stored in saline solution (NaCl 0.9%) at a temperature of 4 degrees Celsius for 30 days. Macroscopic calculus residues, stains and biological remains were removed by manual scraping with periodontal curettes.

Each tooth received four cavity preparations on the buccal (vestibular) surface, performed using a high-speed handpiece with water cooling and a 2,135 diamond bur. A different brand of microbrush was used for each preparation. The shape of the diamond bur defined the cavity design, with the depth standardized to half the height of the bur and the length corresponding to the full active portion. All cavity preparations were carefully inspected to eliminate sharp angles and ensure smooth, well-defined margins.

### 2.2 Adhesive carrying and release capacity

To analyze the loading capacity of the microapplicators, fifteen microapplicators of each brand were used. The microapplicators were then weighed individually on an analytical precision scale and numbered from one to fifteen. Next, a drop of adhesive (Scotchbond™ Universal Adhesive, 3M ESPE, St. Paul, MN, USA) was dispensed into a plastic capsule.

The applicator was immediately submerged in the capsule to be completely covered by the adhesive and at the same time taken to the analytical scale, and its weight was recorded. In this way, it was possible to measure the difference in weight of the microapplicator before receiving the adhesive and after receiving the adhesive, which generates the result of the amount of liquid present in the microapplicator. Immediately afterwards, the microapplicator was rubbed on the cavity preparation for 30 s, in order to release the amount of adhesive absorbed by the active part. The same microapplicator was weighed again to record the final weight.

### 2.3 Optical photography before and after use of microapplicators

A metal device was made to position fifteen new microapplicators before and after use in a standardized manner under the optical microscope, so that the photographs were always taken in the same way. Thus, the microapplicators were removed from the original box, marked with numbers from one to fifteen, and immediately inserted into the device to be photographed. The magnification used was x5 for all microapplicators. After taking the initial image, all microapplicators were carefully stored to be used in the adhesive procedures. After being used in the application of the adhesive system, all fifteen microapplicators from each group were placed back into the device and the final photographs were taken for comparative analysis. Each group was named opi and opf (initial optical and final optical).

### 2.4 Electron microscopy before and after use

For electron microscopy analysis, one microapplicator from each commercial brand was randomly selected. Both a new, unused applicator and one used during the study were chosen to enable comparisons between initial and final conditions, as well as among different brands. The samples were sectioned near the active tip, ensuring the area containing the bristles remained exposed. They were then mounted onto stubs for examination under the scanning electron microscope. The samples were examined under a scanning electron microscope (Zeiss EVO LS10, Carl Zeiss Microscopy GmbH, Jena, Germany) operated at an accelerating voltage of 15 kV in secondary electron detection mode. Images were captured at various magnifications (×70–×110) to evaluate the surface morphology, distribution, and integrity of the bristles before and after use. Images were qualitatively analyzed to identify morphological differences among the different brands and between the unused and used applicators.

### 2.5 Adhesive procedure

For the adhesive procedure technique, the fifteen microapplicators that had been initially photographed were used. Phosphoric acid was applied to the cavosurface angle of the preparation, with this acid remaining restricted to the enamel only. The acid was removed with an air/water spray for 15 s. The preparation was dried with air jets for 15 s. A drop of adhesive was dispensed into a plastic capsule and the microapplicator was immediately dipped into the capsule to absorb the adhesive. Next, the microapplicator was manually agitated in the cavity for 30 s, performing the technique according to the adhesive's own usage protocol. After application, each of the 15 microapplicators was stored individually to be subjected to final optical photography and also to scanning electron microscopy.

### 2.6 Statistical analysis

All quantitative data were analyzed using IBM SPSS Statistics version 25.0 (IBM Corp., Armonk, NY, USA). The Shapiro–Wilk test was applied to assess normality and the Levene test for homogeneity of variances. Since the data met the assumptions of parametric analysis, a one-way analysis of variance (ANOVA) was performed to compare the adhesive carrying and release capacities among the four microapplicator brands. When significant differences were found (*p* < 0.05), Tukey's *post hoc* test was applied for pairwise comparisons.

## 3 Results

### 3.1 Microapplicator carrying capacity

The average results of the carrying capacity and release of adhesive product of each brush are described in the tables below (Table 1).

**Table 1:** Average loading capacity and release of dentin adhesive, for each brand of brush analyzed.

Brand of brush	Adhesive loading capacity	Adhesive release capacity (%)
Angelus	11,51 mg	97,94%
FGM	9,29 mg	96,48
KG Sorensen	11,57 mg	98,02%
SDI	13,5 mg	98,32%

### 3.2 Scanning electron microscopy

Electron microscopy images with 80x magnification of new and used microapplicators can be seen in the figures below (Figures 1–8).

**Figure 1:**
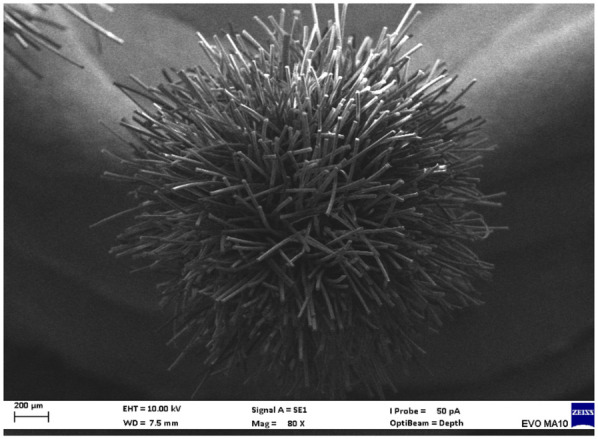
New Angelus microapplicator.

**Figure 2:**
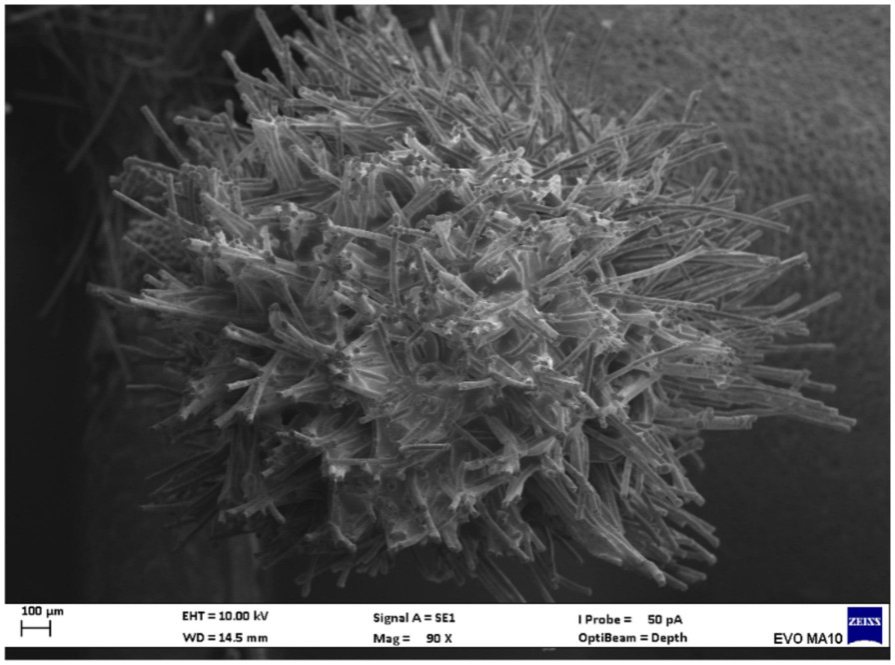
Used Angelus microapplicator.

**Figure 3:**
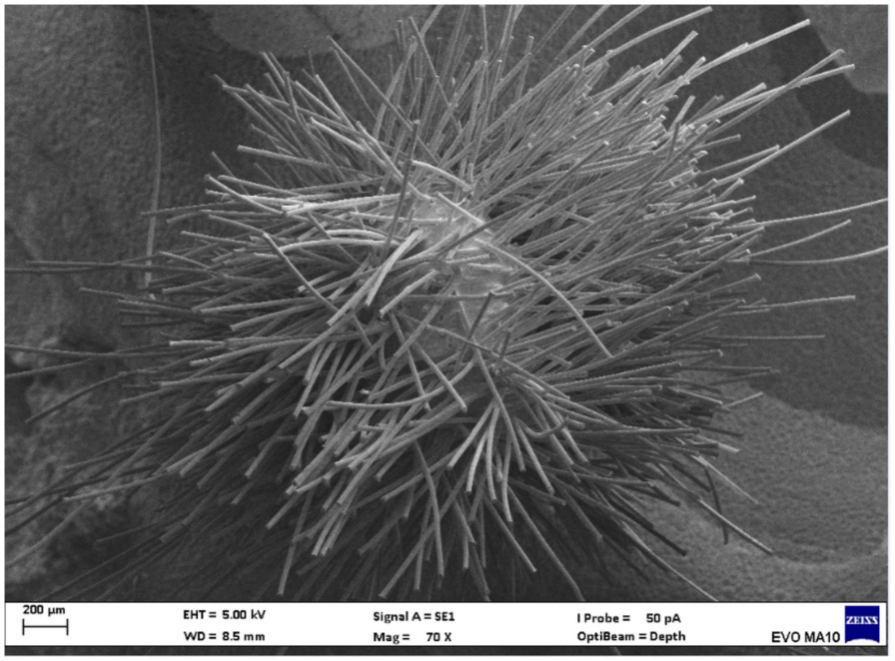
New FGM microapplicator.

**Figure 4:**
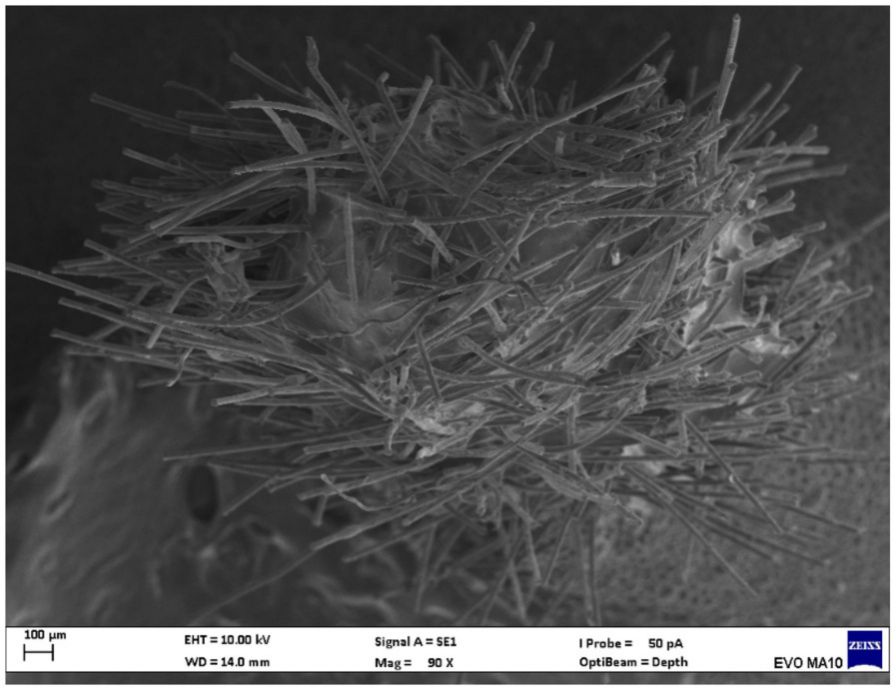
Used FGM microapplicator.

**Figure 5:**
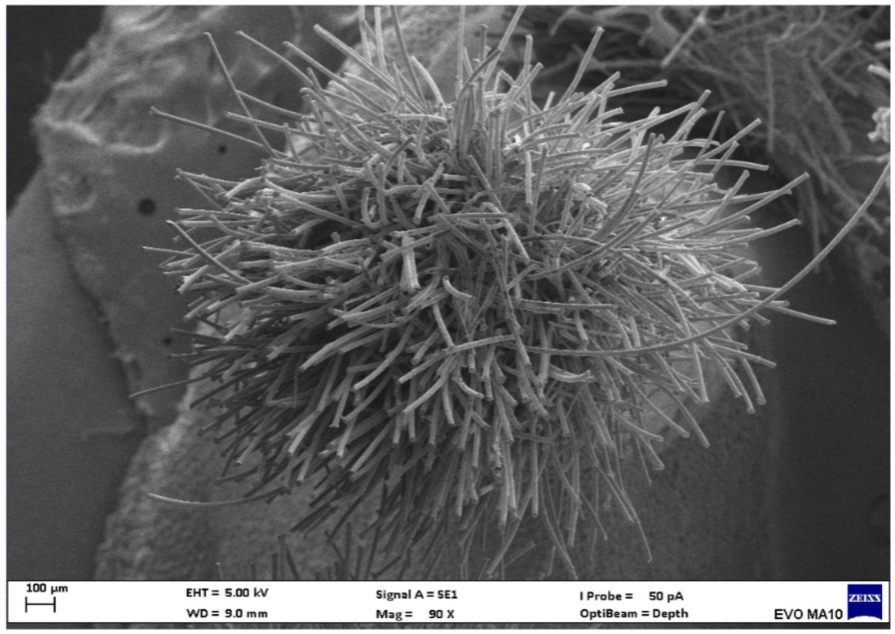
New KG Sorensen microapplicator.

**Figure 6:**
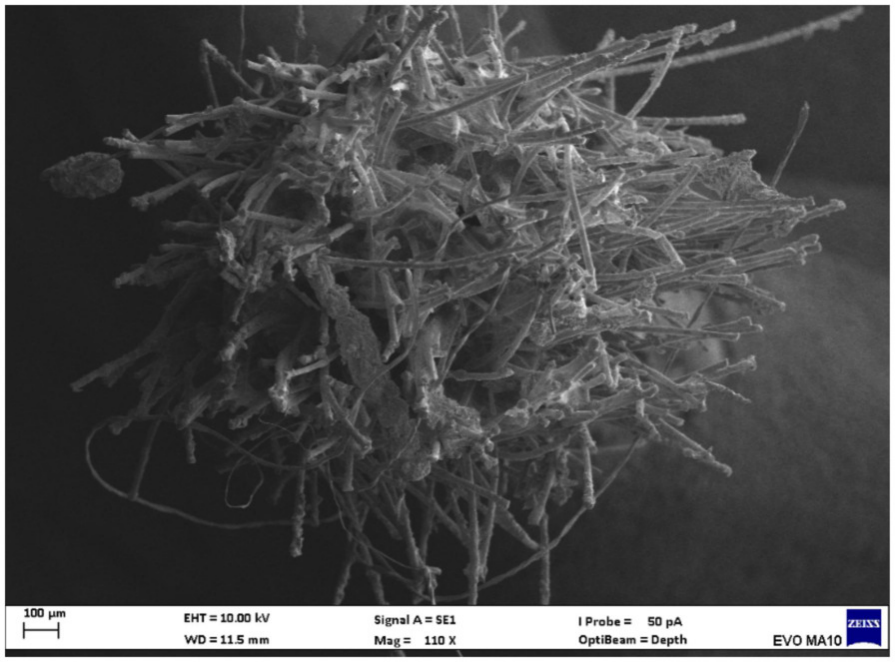
Used KG Sorensen microapplicator.

**Figure 7:**
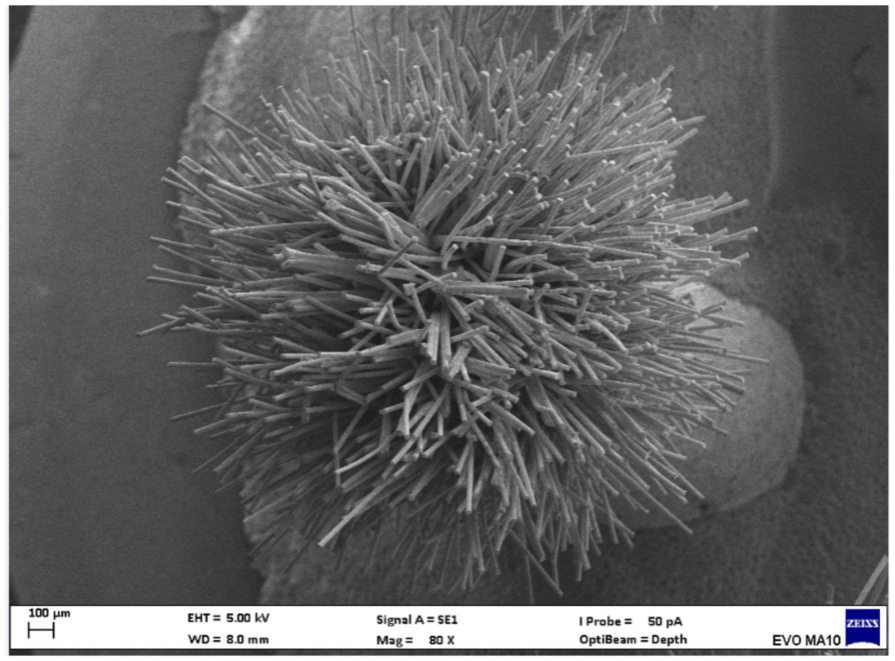
New SDI microapplicator.

**Figure 8:**
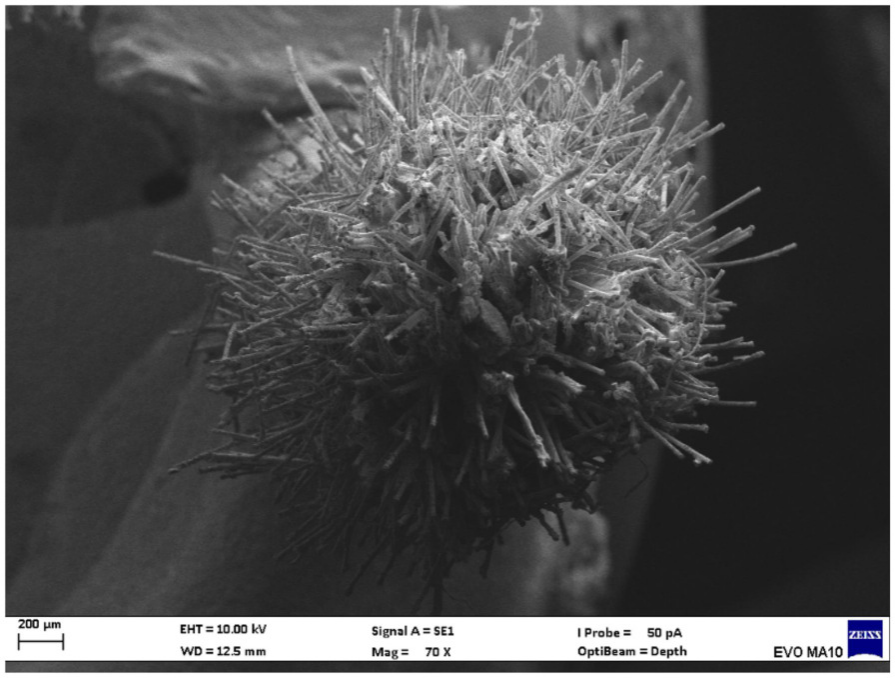
Used SDI microapplicator.

The new microapplicators from the brands Angelus, KG Sorensen and SDI present uniform and homogeneous hairs on the active part, presenting a continuity of hairs throughout the active part. There are no gaps in the active part, in relation to the presence of hairs. Each microapplicator among these three brands mentioned, presents hairs with very similar sizes between them, forming something similar to a “hat” of hairs well distributed over the active part of the microapplicator. The FGM brand presents hairs with different sizes between them. It is also possible to see in the same image, some gaps in the hair cluster, with empty areas without hairs.

Other new microapplicators were used to perform the adhesive procedure on dentin, and then, after use, they were metallized with gold for electron microscopy analysis. It is possible to clearly see that all microapplicators presented deformations after use. The microapplicators of the SDI brand, used, presented the least deformation of the hairs, maintaining the integrity of the spheroidal anatomy of the hair set. The Angelus brand also presented great preservation of the initial anatomy of the hair set, showing little change in structure. The KG Sorensen and FGM brands, however, suffered greater deformation of the hair set, presenting a deformation in its entirety.

### 3.3 Optical microscopy

Optical microscopy images can be seen in the figures below (Figures 9–16).

**Figure 9:**
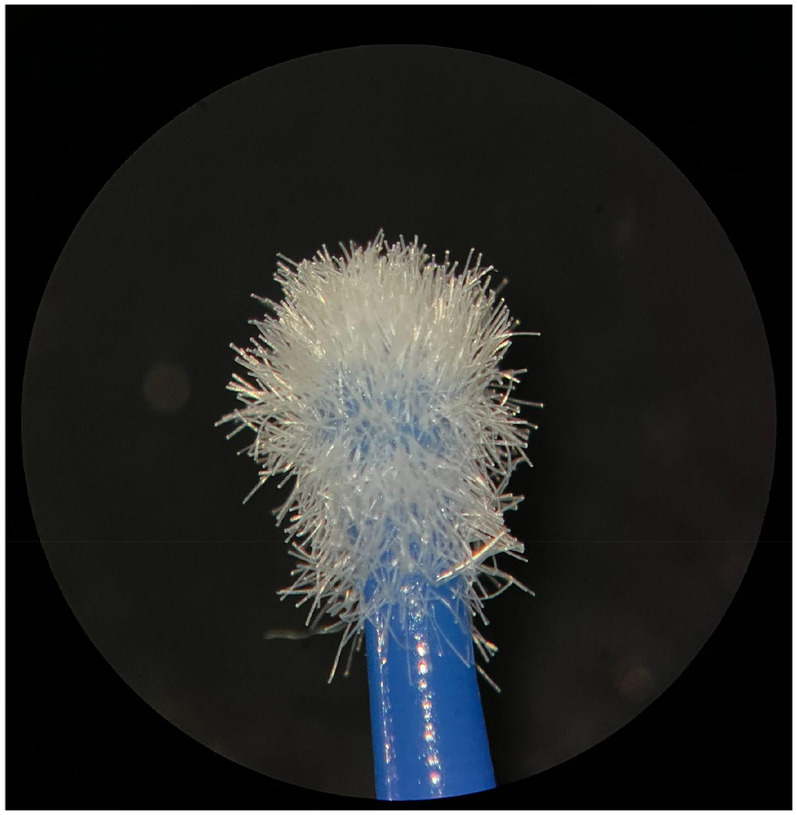
New Angelus microapplicator.

**Figure 10:**
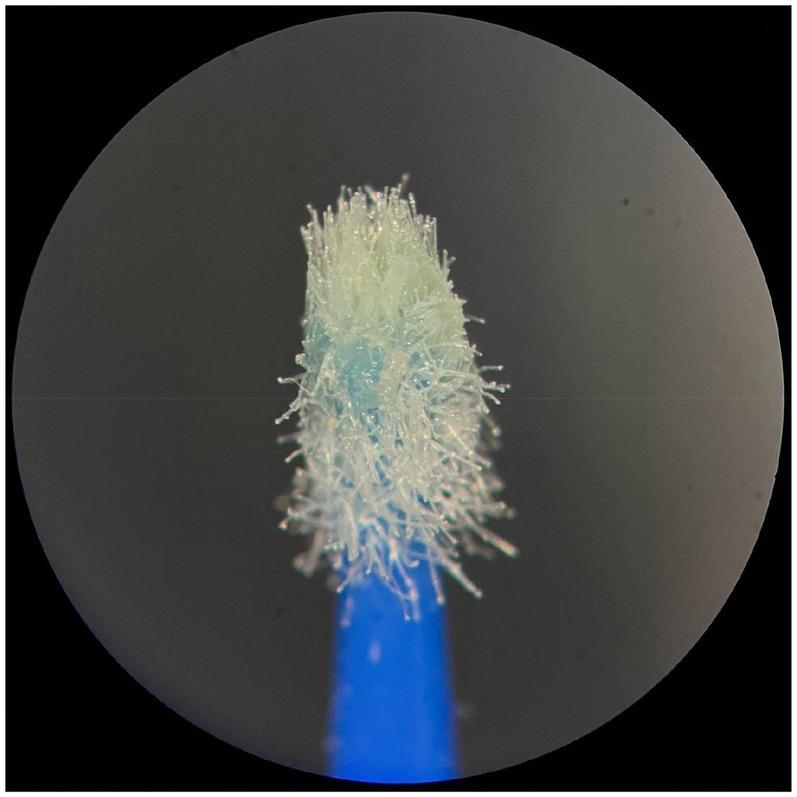
Used Angelus microapplicator.

**Figure 11:**
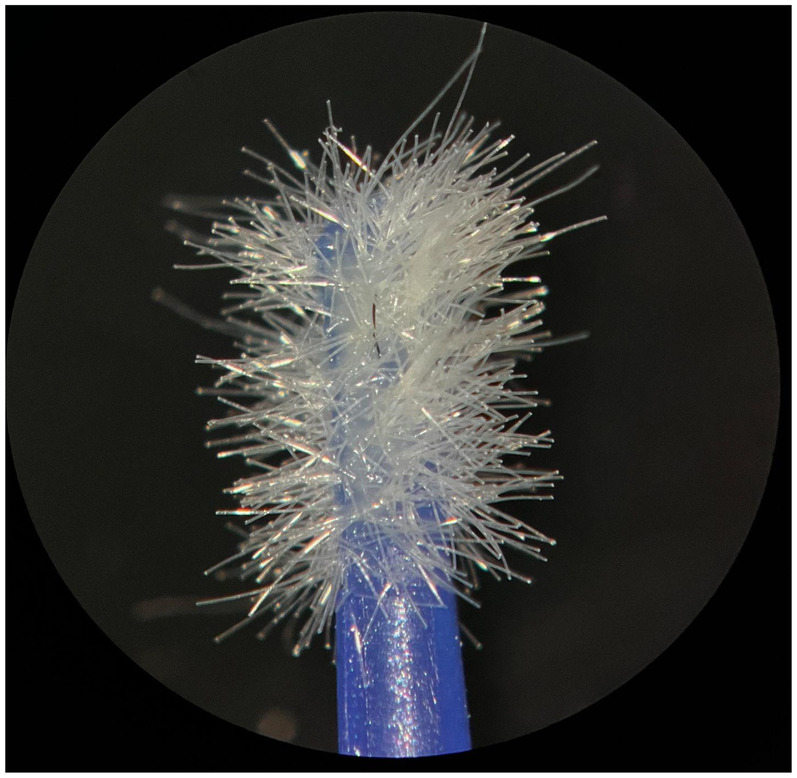
New FGM microapplicator,.

**Figure 12:**
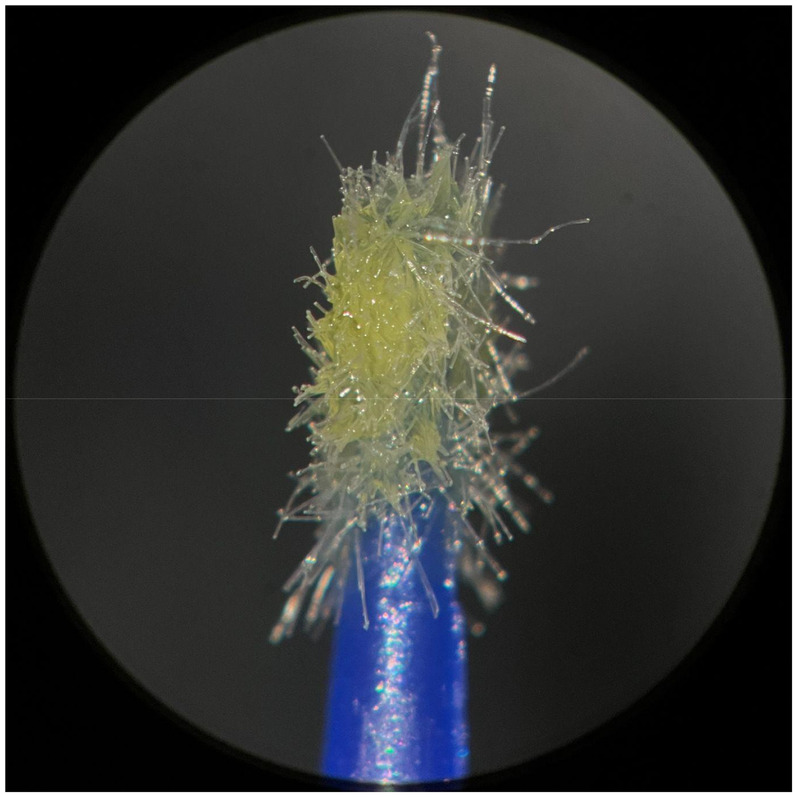
Used FGM microapplicator.

**Figure 13:**
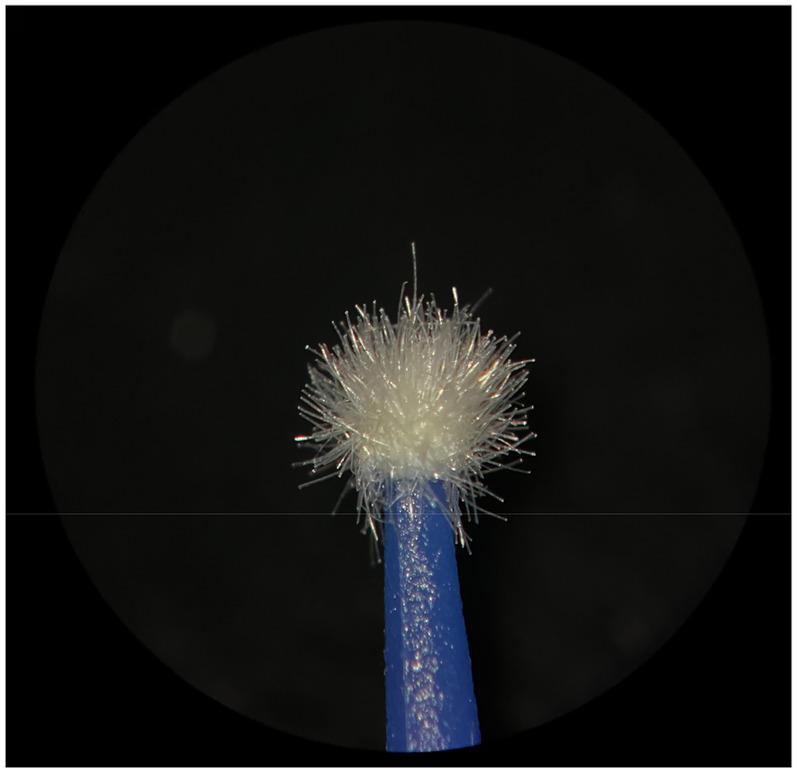
New KG Sorensen microapplicator.

**Figure 14:**
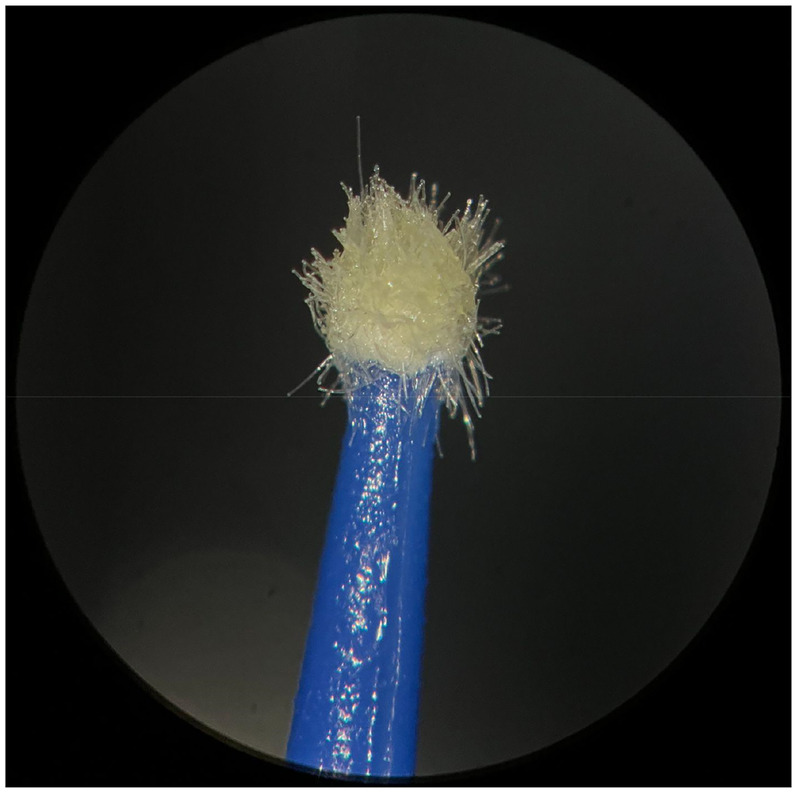
Used KG Sorensen microapplicator.

**Figure 15:**
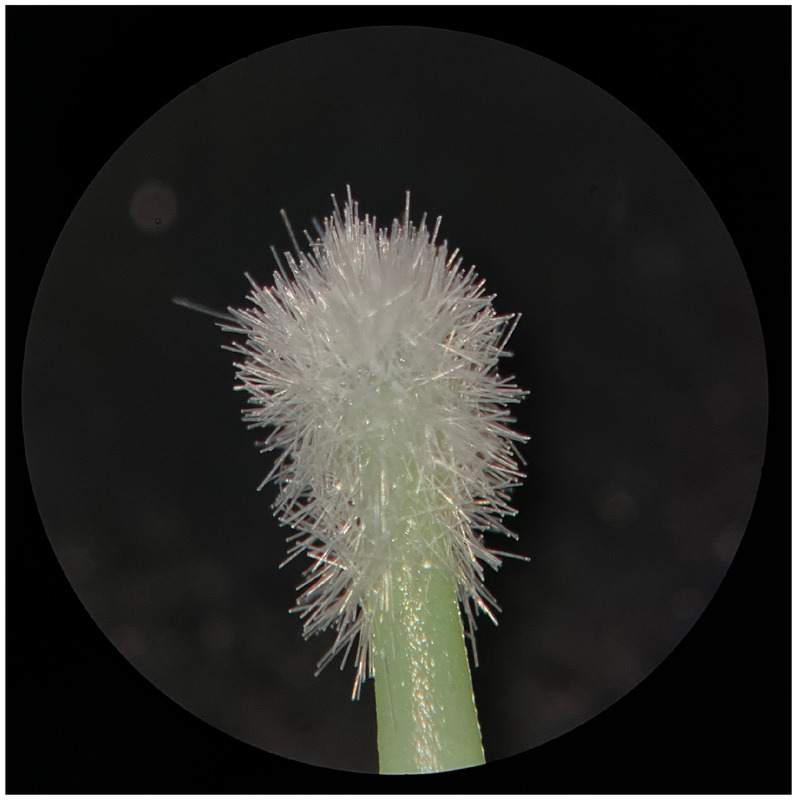
New SDI microapplicator.

**Figure 16:**
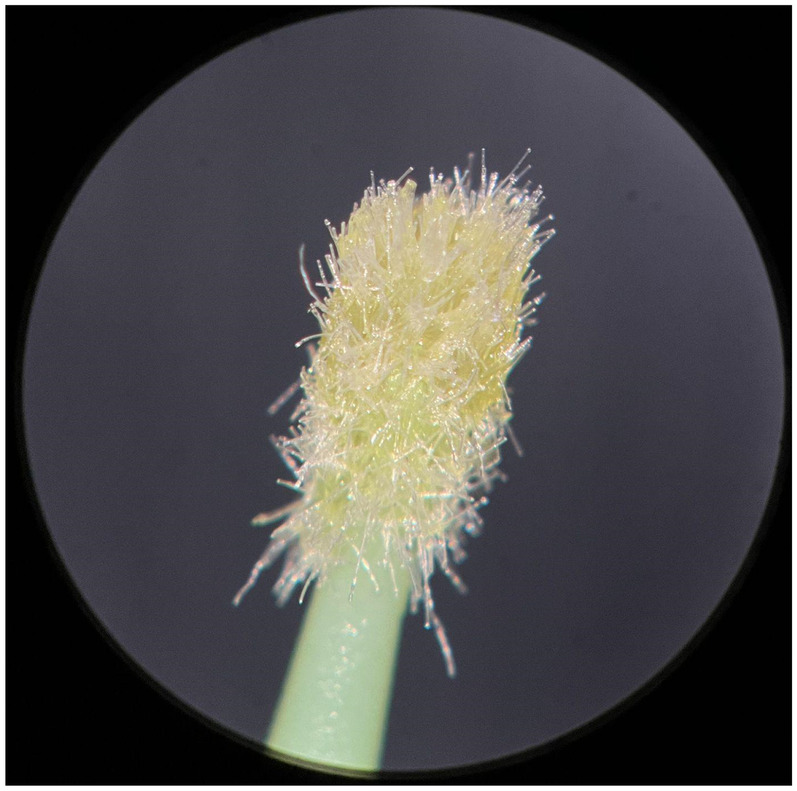
Used SDI microapplicator.

The new microapplicators from the brands Angelus, KG Sorensen and SDI showed good hair distribution in the active part of the microapplicator under optical microscopy, with homogeneity and a circular anatomical shape. Hairs of similar sizes in their distribution and without empty spaces. The microapplicator from the brand FGM, on the other hand, shows flaws in the hair distribution, with empty areas and with little or no hair, as well as irregularity in the size of the hairs.

The same applicators from which the initial optical images were collected received the adhesive system and were applied to the cavities made in the bovine teeth. The SDI microapplicator showed the least deformation of its active part, visually maintaining a large amount of hair. Only compression and condensation of the hairs, due to use. The Angelus microapplicator showed a compressive deformation of the set of hairs but also maintained the anatomy of the active part quite intact, with many hairs present. The KG Sorensen microapplicator showed greater deformation, agglomerating the set of hairs in the center of the active head. The FGM microapplicator also showed deformation of the hairs and the active part, highlighting the areas of hair loss, after its use.

## 4 Discussion

Brush-type microapplicators must have some essential characteristics to be used daily in dental clinics. These characteristics include the ability of the microapplicator to carry the adhesive system in sufficient quantity to be released into the cavity; not losing the adhesive liquid during transport; the ability to release almost all of the adhesive when applied to dentin or enamel; little deformation of its structure when rubbed in the cavity; and no loss of hairs during friction in the cavity (12, 15).

The microscopy images show that the applicators analyzed present quite significant differences between them, mainly in the quantity of hairs and the quality of their positioning. The SDI and Angelus brands have an extremely homogeneous distribution of these hairs, according to the optical and electron microscopy image. This generates confidence for the operator, since the microapplicator can rub the adhesive system practically without losing hairs or becoming deformed. The KG Sorensen microapplicator presents a less homogeneous distribution, followed by the FGM microapplicator, in which the hairs are poorly distributed and with large gaps between them, forming empty spaces.

When analyzing the amount of hair present in the initial image, before use, the SDI and Angelus applicators also visually show more hairs and all very well positioned, which is not the case with the other applicators. This distribution of these hairs should produce a more homogeneous spread of primer and adhesive throughout the cavity, reaching the smallest angles present. The SDI microapplicator, even after use, has a visual characteristic of greater regularity, which conveys greater security to the professional, since it has the visual sensation of “losing” less hairs through friction.

In the scanning electron microscopy images, the SDI and Angelus microapplicators presented very similar regularity and homogeneity of the active part of the applicators, with both hairs having similar sizes for each microapplicator. The KG Sorensen microapplicator also presented regularity of hairs, however, less so than the SDI and Angelus applicators. Some hairs are of different sizes and appear more “loose” on the brush head. The FGM applicator, on the other hand, showed very irregular arrangement of hairs and a really tangled shape, as if it had already been used at some point.

Many dental cavities have small internal angles that are difficult to access, making it difficult for larger diameter micro-applicators to reach them. Even so, when these applicators reach these spaces, they need to be rubbed hard against the dentin (19, 22). At this point, it is important to emphasize how important it is to work with applicators that can correctly deliver the adhesive to the cavity, that do not shed hairs and that remain intact until the end of their use. This research was able to show this, that is, the final state of adhesive micro-applicators: how much they become deformed after being rubbed against the dentin (23).

Microapplicators with adhesively fixed fiber flocks remain the most widely used instruments for applying dental adhesives due to their ease of use and widespread availability. These Conventional Fiber-Flocked Microapplicators (CFFM) typically feature fiber tufts bonded to the plastic handle at the active tip. However, newer technologies have been introduced to overcome limitations such as fiber loss and contamination during adhesive placement. One such innovation is the Fiber-Free Elastomer-Bristle Microapplicator (FEBM), which replaces fiber flocks with flexible elastomer bristles to ensure cleaner, more precise delivery (24, 25).

An example of this technology is ZerofloX™ (MIXPAC Dental, Medmix AG, Baar, Switzerland), an elastomer-bristle microapplicator designed to minimize the risk of contamination and enhance the quality of adhesive application. Its use helps protect the dentin and potentially reduces postoperative sensitivity by delivering adhesive materials uniformly and without bristle residue (25).

The selection of the appropriate microapplicator ultimately depends on the clinical scenario and the operator's judgment (26, 27). Smaller cavities with irregular contours, particularly those requiring the use of advanced adhesive systems, demand more robust applicators that can withstand the force and agitation required for optimal bonding. This active application technique is essential for improving adhesive infiltration and bond strength to dentin (28, 29). In this context, microapplicators from SDI and Angelus demonstrated superior mechanical resistance, followed by those from KG Sorensen and FGM.

Limitations of the study include its *in vitro* design, which may not fully replicate intraoral conditions such as temperature, humidity, and operator variability. In addition, bovine teeth may differ from human teeth in terms of tubule density, enamel prism arrangement, and mineralization pattern, which may influence adhesive interaction and penetration. Moreover, only a limited number of microapplicator brands were evaluated, and long-term effects such as fiber degradation or interaction with various adhesive chemistries were not assessed. Further, only one adhesive system was tested, which limits the extrapolation of results to other adhesive formulations with distinct chemical compositions.

Future directions should include expanded comparative studies incorporating a wider range of applicator designs and adhesive systems under simulated clinical conditions. Investigating the effect of microapplicator design on bond strength, clinical longevity, and the incidence of postoperative sensitivity would provide further insight into optimizing adhesive protocols. Moreover, clinical trials are essential to confirm the laboratory findings and guide evidence-based selection of microapplicators in restorative procedures.

## 5 Conclusions

Using the methodology described and based on the results presented, this research concludes that the SDI brand microapplicators were the ones that presented the least deformations during standardized use, followed by the Angelus, KG Sorensen and FGM brands. The FGM microapplicators visually presented the smallest amount of hairs present, with gaps between them. The adhesive release capacity was similar for all brands, being above 96%.

## Data Availability

The original contributions presented in the study are included in the article/Supplementary Material, further inquiries can be directed to the corresponding author.
